# Disentangling the Mechanisms Shaping the Prokaryotic Communities in a Eutrophic Bay

**DOI:** 10.1128/spectrum.01481-22

**Published:** 2022-05-31

**Authors:** Huajun Zhang, Yi Yan, Tenghui Lin, Weijuan Xie, Jian Hu, Fanrong Hou, Qingxi Han, Xiangyu Zhu, Demin Zhang

**Affiliations:** a State Key Laboratory for Managing Biotic and Chemical Threats to the Quality and Safety of Agro-products, Ningbo University, Ningbo, China; b Key Laboratory of Applied Marine Biotechnology of Department of Education, Ningbo University, Ningbo, China; c Environmental Monitoring Center of Ningbo, Ningbo, China; State Key Laboratory of Mycology, Institute of Microbiology, Chinese Academy of Sciences

**Keywords:** temperature, community assembly, co-occurrence networks, prokaryotic communities, Xiangshan Bay

## Abstract

Eutrophication occurring in coastal bays is prominent in impacting local ecosystem structure and functioning. To understand how coastal bay ecosystem function responds to eutrophication, comprehending the ecological processes associated with microbial community assembly is critical. However, quantifying the contribution of ecological processes to the assembly of prokaryotic communities is still limited in eutrophic waters. Moreover, the influence of these ecological processes on microbial interactions is poorly understood. Here, we examined the assembly processes and co-occurrence patterns of prokaryotic communities in a eutrophic bay using 156 surface seawater samples collected over 12 months. The variation of prokaryotic community compositions (PCCs) could be mainly explained by environmental factors, of which temperature was the most important. Under high environmental heterogeneity conditions in low-temperature seasons, heterogeneous selection was the major assembly process, resulting in high β-diversity and more tightly connected co-occurrence networks. When environmental heterogeneity decreased in high-temperature seasons, drift took over, leading to decline in β-diversity and network associations. Microeukaryotes were found to be important biological factors affecting PCCs. Our results first disentangled the contribution of drift and microbial interactions to the large unexplained variation of prokaryotic communities in eutrophic waters. Furthermore, a new conceptual model linking microbial interactions to ecological processes was proposed under different environmental heterogeneity. Overall, our study sheds new light on the relationship between assembly processes and co-occurrence of prokaryotic communities in eutrophic waters.

**IMPORTANCE** A growing number of studies have examined roles of microbial community assembly in modulating community composition. However, the relationships between community assembly and microbial interactions are not fully understood and rarely tested, especially in eutrophic waters. In this study, we built a conceptual model that links seasonal microbial interactions to ecological processes, which has not been reported before. The model showed that heterogeneous selection plays an important role in driving community assembly during low-temperature seasons, resulting in higher β-diversity and more tightly connected networks. In contrast, drift became a dominant force during high-temperature seasons, leading to declines in the β-diversity and network associations. This model could function as a new framework to predict how prokaryotic communities respond to intensified eutrophication induced by climate change in coastal environment.

## INTRODUCTION

Eutrophication has occurred widely in freshwater and marine ecosystems, resulting in a broad range of ecological and biogeochemical effects (1). In coastal regions, the consequences of anthropogenic nutrient inputs, particularly in shallow and enclosed bodies of water, are progressively leading to toxic algal blooms, hypoxia, and biodiversity loss, all of which are prominent in impacting local ecosystem structure and functioning (2). Prokaryotes can respond quickly to anthropogenic disturbances owing to their unique position in metabolizing organic substrates and nutrient remineralization in biogeochemical cycles (3). Several previous long-term sampling efforts to investigate the main factors affecting the seasonality of marine microbial communities mainly focused on the relative importance of temperature and nutrients (4–7). These are obviously influential, as temperature has a large impact on microbial metabolism (8) and environmental niche partitioning depends on the availability of nutrients in different regions (9). However, factors driving the dynamics of prokaryotic community compositions (PCCs) in eutrophic waters are usually complex and may include the compounding of several factors, making it challenging to disentangle them. Thoroughly elucidating the factors, including community assembly and microbial interactions, that contribute to the seasonality of coastal PCCs in eutrophic waters can lead to more accurate predictive modeling of marine ecosystems in response to eutrophication.

Microbial community assembly is a central topic in revealing the function of the ecosystem (10–13). Two prevalent and complementary ecological processes, neutral- and niche-based theories, are widely applied to elucidate microbial community assembly. The neutral theory predicts that stochastic processes, including the drift, dispersal, and local extinction, shape community assembly (10, 14, 15). Stochastic processes are expected to play crucial roles in determining microbial community compositions (15–17), particularly in early communities with the inherent randomness of dispersal and ecological drift driving composition (10, 18). The niche-based theory declares that deterministic processes caused by biotic and abiotic factors drive microbial assembly, mainly due to diverse niche preferences and the fitness of microbes (19–22). Microbial interactions are generally regarded as deterministic processes (10), but so far, there is still a large gap in how interactions between microeukaryotes and prokaryotes influence PCC variations in eutrophic waters during seasonal changes. Interactions between microbes in complex network systems could be highly affected by these ecological processes (23). For instance, selection pressure imposed by environmental factors can drive highly associated microbes to group together or generate modules in co-occurrence networks in response to specific environmental conditions (24). Co-occurrence networks are widely regarded as an effective tool with which to infer microbial interactions. Using this approach, intrinsic interactions (including grazing, mutualism, symbiosis, cross-feeding, and parasitism) can be clearly elucidated (25). Although correlations in the network do not accurately represent true interactions between microbes, network analyses can still help us to acquire and elucidate information on highly diverse communities.

So far, studies on the assembly mechanisms and co-occurrence patterns of PCCs in eutrophic waters during the seasonal transitions are still limited. Xiangshan Bay, a semienclosed bay (approximately 70 km long, 3–8 km wide, and 10 m deep on average) in Zhejiang, China, is an important aquacultural region with limited water exchange ability and severe eutrophication (19). Moreover, Xiangshan Bay is vulnerable to several anthropogenic disturbances caused by tourism, industry, and agriculture. In such fluctuating environments, predictable patterns of assembly and the co-occurrence of microbial communities are particularly important for understanding the mechanisms that preserve ecosystem stability. In this study, based on samples collected monthly throughout the year in Xiangshan Bay, we tried to demonstrate the assembly processes and co-occurrence patterns of PCCs under eutrophic conditions. Therefore, the present study was designed to resolve the following critical issues: (i) How do ecological processes affect the seasonality of PCCs? (ii) What seasonal trends emerge in the intradomain co-occurrence networks of the PCCs? (iii) What is the relationship between ecological processes and co-occurrence networks in eutrophic waters? By answering these questions, our findings will provide the largest inventory so far of the mechanisms shaping the seasonality of prokaryotic communities in this eutrophic bay.

## RESULTS

### Seasonal patterns of the environmental factors.

Surface seawater temperature was high in summer (average of 27.3°C) and autumn (average of 25.0°C, Fig. S2). Salinity ranged between 21.6 and 28.9 psu with the lowest value in summer. The dissolved oxygen (DO) was significantly covariant with temperature and was thus disregarded in the following analyses. Changes in pH (7.81–8.16), nitrate (0.352–0.943 mg/L), and silicate (0.587–1.61 mg/L) showed similar trends, declining from winter to summer and increasing from summer to autumn. Nitrite, with the highest concentration in summer (average, 0.031 mg/L), showed the opposite trend as nitrate. Chemical oxygen demand (COD; 0.230-1.30 mg/L) and phosphate (0.024–0.091 mg/L) decreased from winter to spring and then increased from spring to autumn. Chlorophyll a (Chl a) and ammonium levels were relatively homogeneous throughout the year. The average environmental heterogeneity was the highest in winter (0.468) then decreased to 0.343 in summer as the temperature increased (Fig. S2 in the supplemental material).

### Patterns of prokaryotic community compositions.

The dominant phyla in the Xiangshan Bay were *Proteobacteria*, *Bacteroidetes*, *Thaumarchaeota*, and *Actinobacteria* (Fig. S3). The most abundant classes were *Alphaproteobacteria*, *Gammaproteobacteria*, *Thaumarchaeota* Marine Group I (MGI), *Flavobacteriia*, and *Betaproteobacteria* (Fig. S4, Table S1). Interestingly, relative abundance of *Cyanobacteria* was much higher during high-temperature seasons, especially in summer (9.60%, Table S1). Temperature was strongly and positively associated with *Cyanobacteria* (Spearman’s ρ = 0.875, *P < *0.001), SAR406 (ρ = 0.684, *P < *0.001), *Euryarchaeota* (ρ = 0.536, *P < *0.001), and *Actinobacteria* (ρ = 0.429, *P < *0.001) but negatively correlated with *Proteobacteria* (ρ = −0.517, *P < *0.001) and *Verrucomicrobia* (ρ = −0.370, *P < *0.001) abundances (Fig. S3).

**FIG 1:**
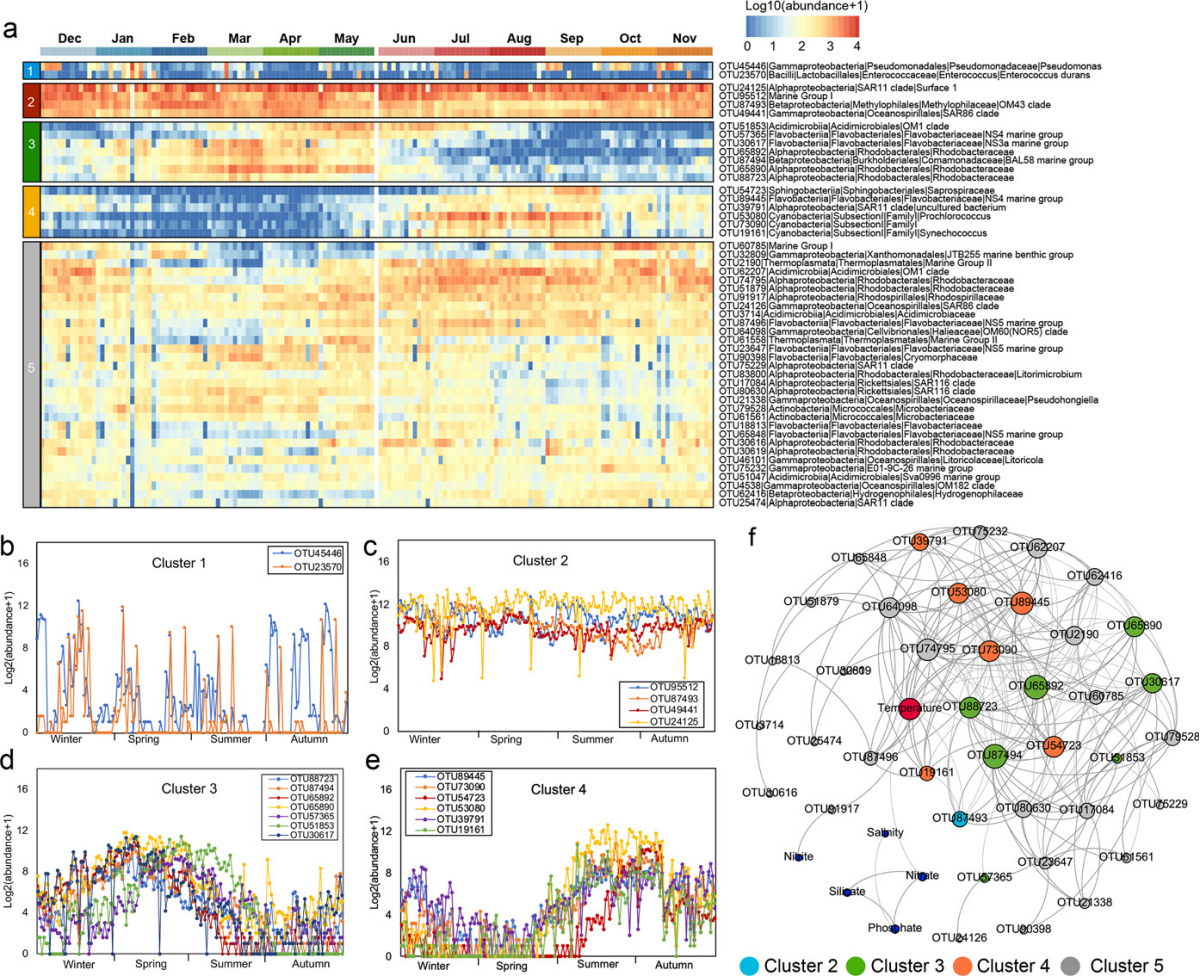
Seasonal patterns of the 50 most abundant OTUs. (a) The OTUs were grouped into five clusters according to their patterns of abundance. (b–e) Plots showing the temporal dynamics of Clusters 1 to 4, which were visually assigned to opportunistic (b), ubiquitous (c), spring-associated (d), and summer-associated (e) groups, respectively. (f) Spearman’s rank correlation network with significant (FDR-adjusted *P* value < 0.01) and robust (Spearman’s |ρ| > 0.6) correlations showing the relationship between five clusters and environmental factors.

Based on visual similarities in the temporal patterns, we assigned the 50 most abundant operational taxonomic units (OTUs) to five clusters (Fig. 1a), which were named as follows: opportunistic (Cluster 1), ubiquitous (Cluster 2), spring-associated (Cluster 3), summer-associated (Cluster 4), and nonrelevant (Cluster 5). The spring-associated group contained mainly *Rhodobacteraceae* and *Flavobacteriaceae* (Fig. 1d). *Synechococcus* and *Prochlorococcus* were assigned to the summer-associated cluster (Fig. 1e). However, 31 of the 50 most abundant OTUs were non-seasonal association. Based on network analysis (Fig. 1f), almost all OTUs in the spring- and summer-associated groups correlated with temperature.

### Relevance of temperature and microeukaryotes to PCC variations.

**FIG 2:**
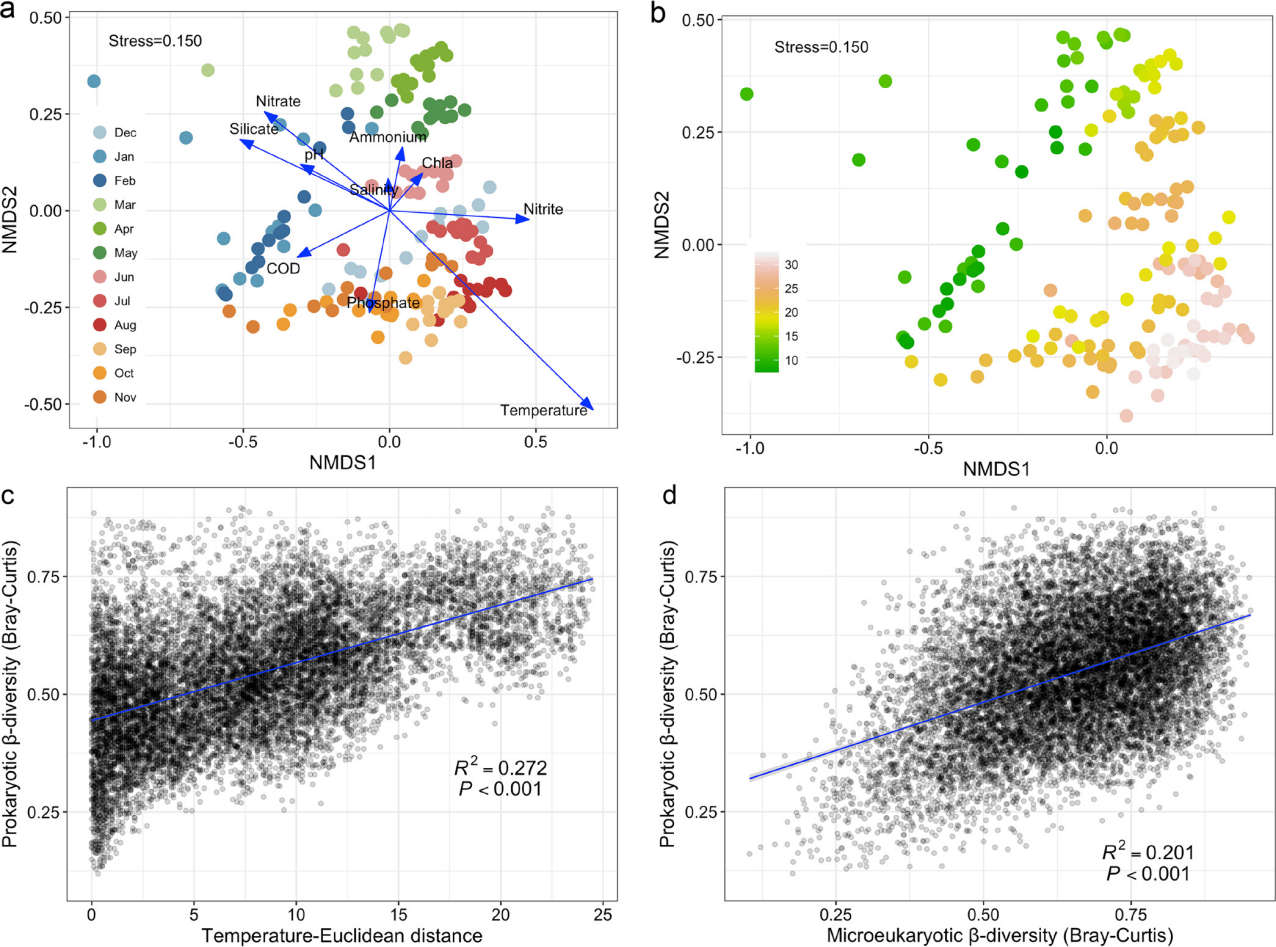
Factors controlling prokaryotic β-diversity. (a) Bray-Curtis distance-based nonmetric multidimensional scaling (NMDS) plot. Arrows indicate environmental factors that were strongly linked to community variations. Each cycle is colored according to its sampling time. Chl a, chlorophyll a content; COD, chemical oxygen demand. (b) Effects of temperature on the prokaryotic β-diversity. The color gradient of each cycle indicates temperature throughout the year. (c–d) Correlations of prokaryotic β-diversity with temperature (Euclidean distance, c) and microeukaryotic β-diversity (Bray-Curtis distance, d).

**FIG 3:**
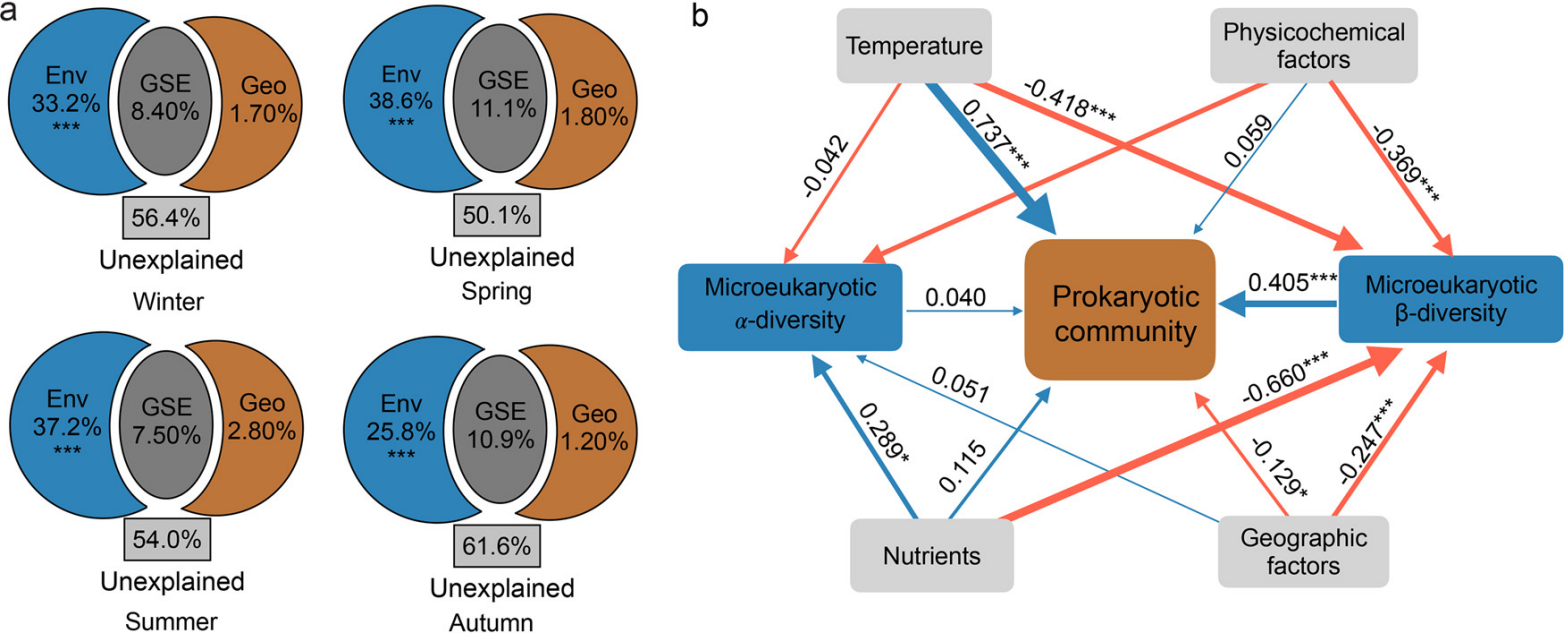
Disentangling the contributions of environmental factors and microeukaryotes to community variations. (a) Variation partitioning analysis showing relative contributions of environmental (Env) and geographic (Geo) factors to community variations. GSE, geographic structured environment factor. (b) Partial least-squares path model showing the relationships among prokaryotic community compositions (PCCs), environmental factors, microeukaryotic community compositions, and geographic factors. PCCs are represented by NMDS2 from the Bray-Curtis distance-based NMDS analysis. Microeukaryotic α-diversity includes richness, Shannon index, evenness, and phylogenetic diversity. Microeukaryotic β-diversity is NMDS1 from the Bray-Curtis distance-based NMDS analysis. Nutrients include ammonium, nitrite, nitrate, phosphate, and silicate. Physiochemical factors consist of pH, salinity, chemical oxygen demand, dissolved oxygen, and chlorophyll a content. Geographic factors include sampling latitude and longitude. The standard path coefficients (*β*) are the numbers near the pathway arrows and indicated by the width and color of arrows, with red and blue arrows representing significant (*P < *0.05) negative and positive pathways, respectively. The goodness of fit value was 0.452.

The overall composition of the prokaryotes substantially differed (MRPP and PERMANOVA, *P < *0.001) when any two seasons were compared (Table S2). Such seasonal community patterns were also clear in the NMDS plot (Fig. 2a), which obviously divided samples into groups depending on seasons with relatively low or high temperatures (Fig. 2b). Moreover, we found that the PCC variations were highly linked with temperature gradients (*R*^2^ = 0.272, *P < *0.001; Fig. 2c), indicating that temperature was the main abiotic factor driving PCC variations across four seasons. Environmental factors significantly (*P < *0.001) explained 33.2%, 38.6%, 37.2%, and 25.8% of the PCC variations in winter, spring, summer, and autumn, respectively (Fig. 3a). The detected geographic factors had weak correlations with variations of PCCs in the four seasons. However, 56.4%, 50.1%, 54.0%, and 61.6% of the variations were unexplained from winter to autumn (Fig. 3a). Variations of the PCCs were strongly correlated (all *P < *0.001) with temperature and the microeukaryotic community across the four seasons (Table S3). Temperature had the largest direct influence on community variations (standardized path coefficient, *β* = 0.737, *P < *0.001, Fig. 3b). Microeukaryotes were the second most powerful factor (*β* = 0.405, *P < *0.001, Fig. 3b), while nutrients and other physicochemical factors did not directly affect PCC variations. We also found that microeukaryotic β-diversity fitted well to the prokaryotic β-diversity (R^2^ = 0.201, *P < *0.001) (Fig. 2d, Fig. S5). These results corroborated the hypothesis that temperature and microeukaryotes were the important respective abiotic and biotic factors shaping the seasonal dynamics of PCCs in this eutrophic bay.

### Assembly processes of prokaryotic community.

**FIG 4:**
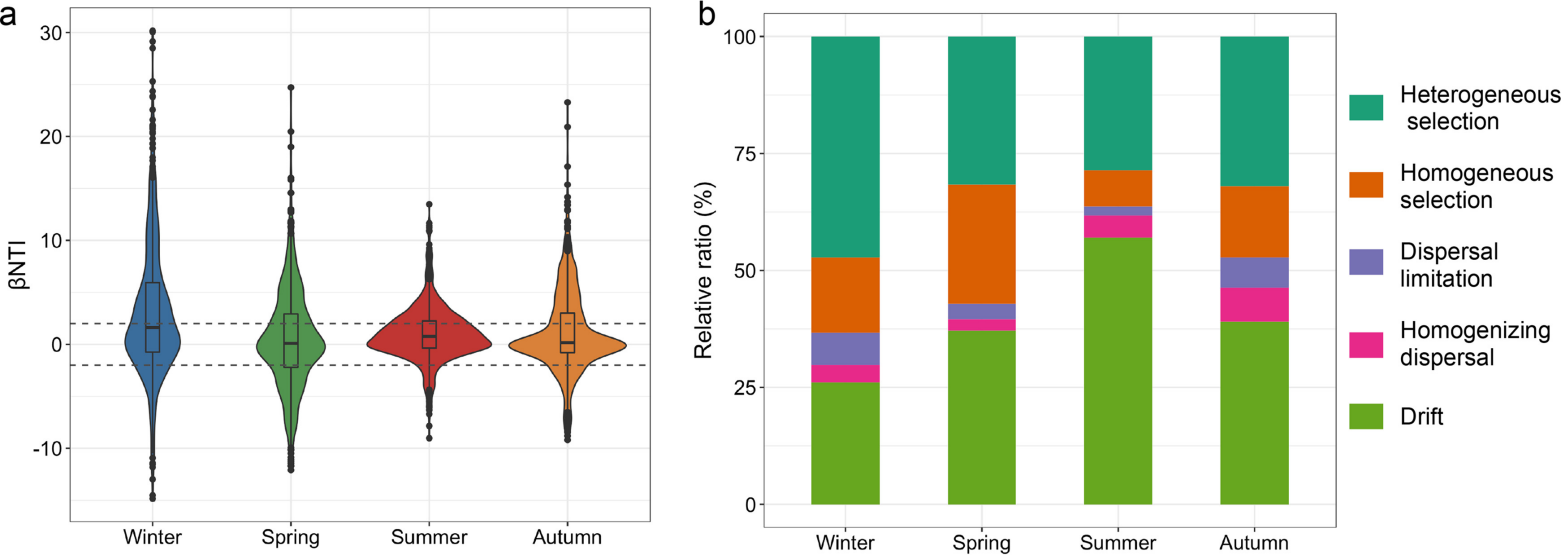
Prokaryotic community assembly processes. (a) Violin plots and boxplots showing distribution of βNTI values in four seasons. Horizontal dashed lines indicate βNTI thresholds at −2 and +2. (b) Contribution of individual ecological processes to prokaryotic community assembly.

The βNTI, together with the RC_bray_ analysis, demonstrated that selection was the dominant ecological process in all seasons except summer, when drift played an overwhelming role in community assembly (Fig. 4a and b). Heterogeneous selection had a major role in defining the community assembly in winter and spring (47.2% and 31.6%, respectively), while homogeneous selection contributed 16.1% and 25.5% of community assembly. Despite not being the dominant process, the proportion of drift still reached 26.1% and 37.1% in winter and spring, respectively. The contributions of heterogeneous and homogeneous selection decreased in the summer (28.6% and 7.7% of the overall community assembly, respectively), while drift reached its maximum proportion (57.0%) (Fig. 4b). Even though the roles of heterogeneous and homogeneous selection increased again in autumn, drift still constituted a significant percentage (39.1%). Nonselective processes, such as homogenizing dispersal and dispersal limitation, had minor influences during all four seasons (Fig. 4b).

### Different patterns of species interactions in the four seasons.

**FIG 5:**
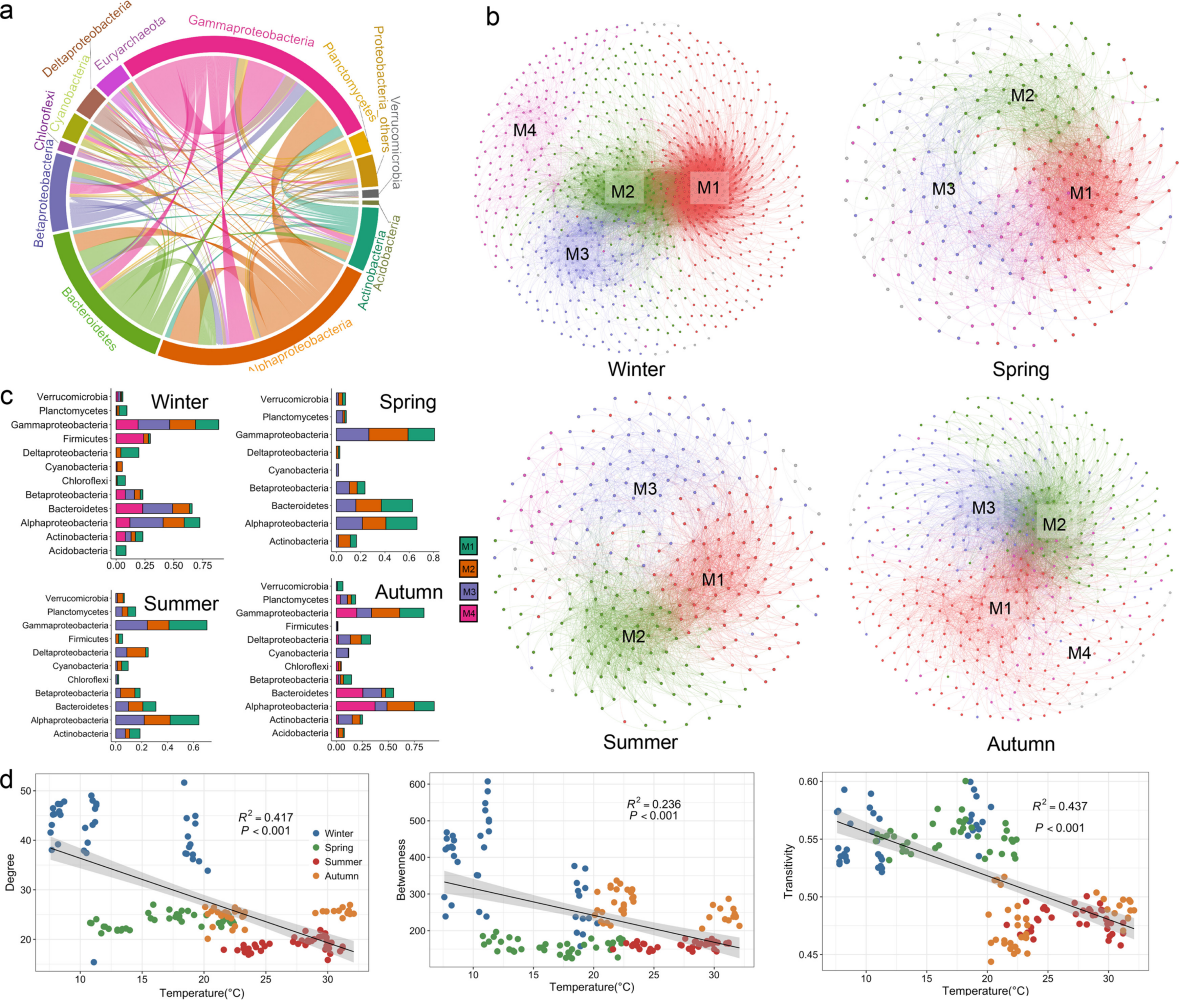
Prokaryotic co-occurrence patterns. (a) Overview of co-occurrence network over four seasons. Connections are colored according to the phylum or class name. (b) Co-occurrence network for four seasons. Nodes are colored by network modules, which were named M1 to M4 by modular weight. (c) Compositions of network modules for four seasons. (d) Relationship between temperature and degree, betweenness centrality, and transitivity centrality.

All four networks fitted well with the power-law model, with R^2^ values of 0.736, 0.662, 0.697, and 0.775 for winter, spring, summer, and autumn, respectively, suggesting that the four networks were scale-free and nonrandom networks (Fig. S6). The prokaryotic meta-network taxa mainly consisted of *Gammaproteobacteria* (22.5%), *Alphaproteobacteria* (21.7%), and *Bacteroides* (14.2%) (Fig. 5a). The connections within their own class were stronger than connections to other classes, indicating that phylogenetic-related taxa co-occurred more frequently.

Number of nodes and edges in the co-occurrence networks varied throughout four seasons, with both being the highest in winter: 855 nodes linked by 17,790 edges (Table S4, Fig. 5b). The network structure in winter was likewise more complex and tighter, as shown by a greater clustering coefficient. Interestingly, we found that connectedness, as measured by the clustering coefficient and average degrees of the networks, appeared to be lower when drift was the dominant factor and greater when selection, particularly heterogeneous selection, became more important (Fig. 4 and 5). In autumn, the co-occurrence network contained 470 nodes and 5,498 edges, which were also significantly higher than those in spring and summer (Table S4, Fig. 5b). There were four clearly defined modules in winter and autumn, and three modules in spring and summer (Fig. 5b). *Gammaproteobacteria*, *Alphaproteobacteria*, and *Bacteroides* were the dominant taxa in all modules (Fig. 5c). Temperature was significantly negatively correlated with three tested features, degree (*R*^2^ = 0.417, *P < *0.001), betweenness centrality (*R*^2^ = 0.417, *P < *0.001), and transitivity (*R*^2^ = 0.417, *P < *0.001), indicating that temperature strongly structured the prokaryotic interactions. The largest number of keystone species were found within the co-occurrence networks in winter (114 OTUs) (Fig. S7), and they were mainly identified as *Alphaproteobacteria* (25 OTUs), *Gammaproteobacteria* (25 OTUs), and Deltaproteobacteria (12 OTUs). There were 0, 2, and 13 keystone species in spring, summer, and autumn, respectively.

Based on the bipartite network for prokaryotes and microeukaryotes, *Chlorophyta* (384 edges) and *Diatomea* (382 edges) appeared to have the greatest number of associations with prokaryotic orders, followed by *Syndiniales* (183 edges) and *Cryptomonadales* (168 edges) (Fig. S8, Table S5). *Flaveobacteriales* had 273 connections with microeukaryotes, including 69 connections with *Chlorophyta* and 66 with *Diatomea* (Fig. S8, Table S5). *Rhodobacterales* and *Oceanospirillales* also frequently interacted with *Chlorophyta* and *Diatomea.* These results indicated that, within all microeukaryotes, *Chlorophyta* and *Diatomea* were the main contributors to PCC dynamics in this eutrophic bay.

## DISCUSSION

Xiangshan Bay is a highly productive ecosystem with complicated physicochemical gradients making it sensitive to fluctuating environmental conditions. As a buffer zone, Xiangshan Bay acts either as an organic matter sink or a reservoir capable of exporting inorganic and organic nutrients to the adjacent sea. In addition to hydrological and physical factors, the degradation and consuming processes carried out by marine microbes determine the balance between accumulation and export, emphasizing the necessity to investigate the seasonal dynamics and assemblages of microbial community. Our findings provide the most comprehensive inventory of prokaryotic community assembly mechanisms and co-occurrence patterns in this eutrophic bay to date.

### Seasonal variations of prokaryotic community compositions.

*Alphaproteobacteria* and *Gammaproteobacteria* dominated the PCCs in Xiangshan Bay throughout the year (Fig. S4, Table S1). The prevalence of *Flavobacteria* in spring (Fig. S4, Table S1) coincided with a rise in nutrient-rich conditions, showing that this taxon prefers more of a productive environment (26). *Cyanobacteria* was obvious summer-associated taxa in this eutrophic bay. Previous research showed that warming caused an increase in the abundance and biomass of *Cyanobacteria* in eutrophic waters (9, 27), which is consistent with our findings (Fig. S4, Table S1). The predominance of *Cyanobacteria* in summer was largely due to *Synechococcus* and *Prochlorococcus* (Fig. 1). *Synechococcus* is found in nearly all marine habitats, whereas *Prochlorococcus* is limited to warmer oligotrophic seas and is absent from colder nutrient-rich waters (28). However, *Prochlorococcus* can proliferate in Xiangshan Bay with serious eutrophication (Fig. 1), which contrasts with the traditional description of their habitat. Moreover, an investigation of phytoplankton abundance in the East China Sea also reported an absence of *Prochlorococcus* along the coastal shoreline west of 124°E (29). It is possible that *Prochlorococcus* would have been detected if the researchers used the more precise amplicon sequencing method. As a productive agricultural bay, Xiangshan Bay contains a large amount of animal waste, which probably led to the high abundance of Enterococcus durans, reminding us that intensive anthropogenic pollution has a profound influence on the marine environment.

Association network analysis revealed that spring- and summer-associated taxa were significantly correlated with temperature (Fig. 1d and e). Temperature is critical in structuring microbial communities over space (6, 30) and time (4, 31). Aside from being associated with temperature, the seasonal taxa showed distinct persistence periods that may also be attributed to various ecological strategies. The abundance dynamics of the spring- and summer-associated taxa may indicate a high growth rate when suitable resources are available, and a rapid decline due to highly competitive pressure and predation. Furthermore, the presence of ubiquitous taxa may reflect a lower amount of pressure from competition or predation, allowing these taxa to persist longer in the ecosystem. The taxa classified as opportunistic OTUs may occasionally increase in abundance when triggered by suitable environmental conditions.

### The community assembly was mediated by a balance between deterministic and stochastic processes.

**FIG 6:**
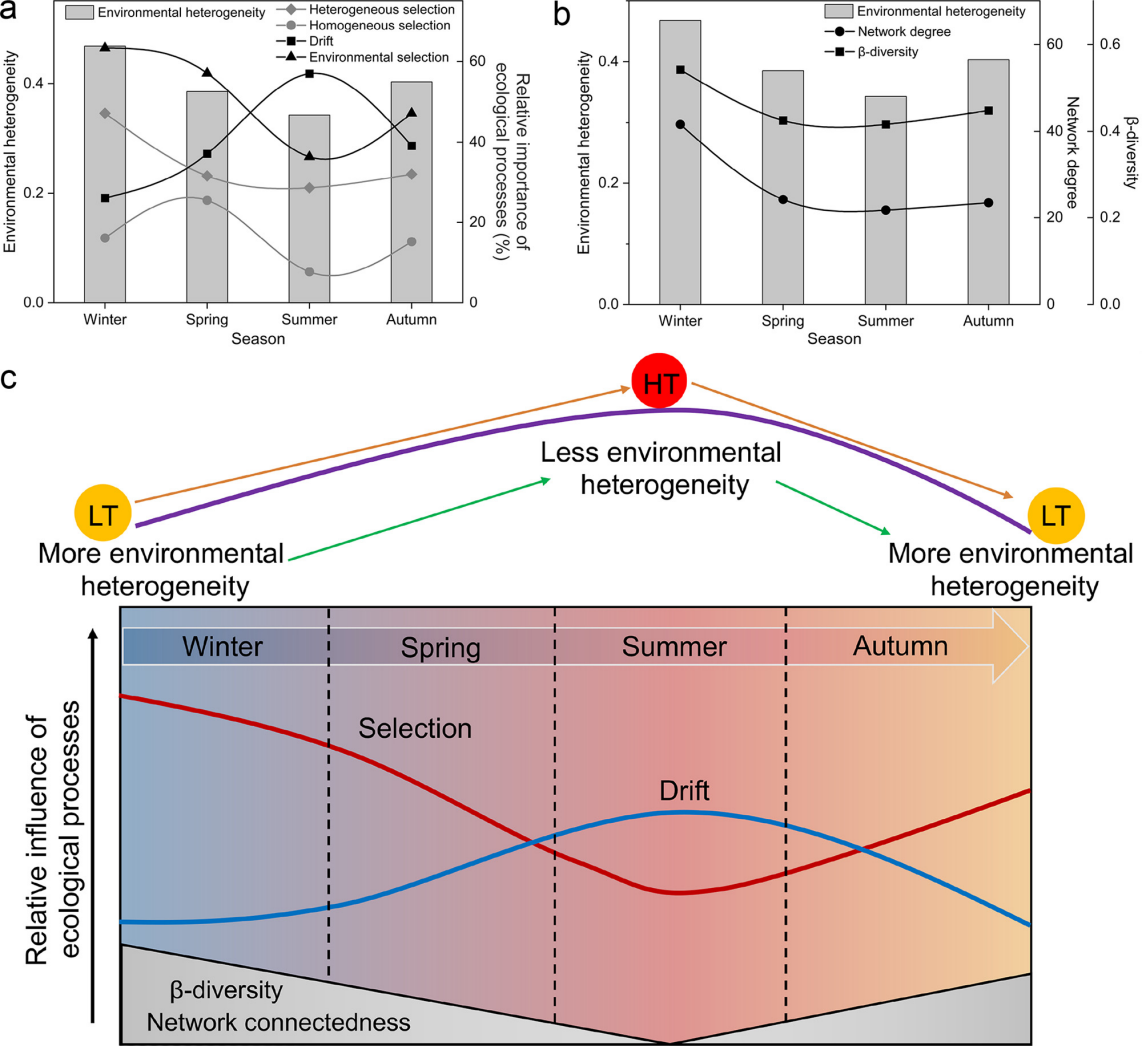
Conceptual model revealing how temperature and environmental heterogeneity affect ecological processes. (a) Evidence showing the patterns of selection and drift in four seasons with changes in environmental heterogeneity, supporting the conceptual model (c). (b) Evidence showing the patterns of β-diversity and microbial interactions, supporting the conceptual model (c). LT, low temperature; HT, high temperature; red and blue lines represent trends of environmental selection and drift process, respectively.

Variation partitioning approach (VPA) results showed that environmental factors can obviously explain the community variation over the four seasons (Fig. 3a). Thus, we proposed that environmental selection could be a primary driver of prokaryotic community assembly in this eutrophic bay. Congruently, in the East China Sea, selection was also found to be more significant in shaping bacterial communities in surface seawater (32). Our results likewise revealed that selection was the primary assembly process when environmental heterogeneity was high (Fig. 4). Previous studies demonstrated that temperature is the major influential factor imposing selection pressure on prokaryotes (33–36), but this literature mainly tried to elucidate the community assembly from the spatial aspect. Therefore, we expanded the information in the literature to include both spatial and seasonal variability in this eutrophic bay. Firstly, we found that, with high environmental heterogeneity (winter, spring, and autumn), PCCs were mostly influenced by selection (Fig. 6a). In this situation, selection pressures in each local community may effectively filter species according to their fitness, resulting in a high β-diversity (Fig. 6b), whereas nonselective processes had less importance. Of the selection processes, heterogeneous selection was more significant than homogeneous selection in structuring PCCs (Fig. 4, 6a). Heterogeneous selection should predominate when environmental factors change in irregular patterns, producing a high compositional turnover. In contrast, homogeneous selection was mainly induced by environmental factors that were relatively predictable. Secondly, with low environmental heterogeneity (i.e., summer), drift was found to be the main ecological force driving PCCs, and it is perhaps the sole equivocally stochastic mechanism in nature (10), affecting species abundance through random births and deaths. Drift is an important factor contributing to a large proportion of the unexplained variance in VPA (10). Until now, the contribution of drift to stochasticity in bacterial communities with high species abundance is still unknown (10). Because of the random recolonization of certain taxa after escaping the strong tide perturbation in Xiangshan bay throughout the summer (37), drift mainly resulted from species death and extinctions (38). *Gammaproteobacteria* are the dominant taxa in Xiangshan Bay, and they comprise numerous opportunists who are r strategists with high maximal growth rates in a favorable environment (39). A previous study suggested that the dominants of r strategists can enhance the contribution of drift on community assembly (39). Moreover, it had been suggested that drift may be particularly significant for the rare taxa in environments with high bacterial densities (40). Drift can lead to a significant gain in β-diversity and loss of α-diversity, especially in fluctuating environments and where dispersion is limited (41). Therefore, the turnover rates for prokaryotes in Xiangshan Bay may be faster than what we supposed under eutrophic conditions.

It should be noted that, even when selection was dominant, the role of drift cannot be neglected (Fig. 4). In other words, both the deterministic processes represented by selection and the stochastic processes represented by drift cannot occupy absolute roles in structuring PCCs. Thus, we proposed that PCCs in Xiangshan Bay are driven by a balance between deterministic and stochastic processes, with the former being more important during cold seasons and the latter during warm seasons (Fig. 6c). It has been proposed that deterministic and stochastic processes are controlled by general principles throughout ecosystems (42). In addition, we speculated that selection is the most powerful ecological process shaping PCCs during low-temperature seasons, but the role of selection decreases during high-temperature seasons, allowing stochastic processes such as drift to become more prevalent. As indicated by the combined results from VPA and βNTI, the relatively minor geographic effect on communities indicated a weak influence of dispersal limitation and homogenizing dispersal on PCC variations in Xiangshan Bay. Dispersal limitation is significantly related to geographic scale and microbial cell size, and homogenizing dispersal is similar to source-sink dynamics and mass effect (10, 43). Thus, at small geographic scales and low mass effect conditions, such as those seen in Xiangshan Bay, dispersal limitation and homogenizing dispersal are found to have a minor impact on the prokaryotic community.

### Relationships between community assembly and interaction patterns.

Determining the action of ecological processes on species interactions is fundamental for understanding the mechanisms supporting seasonal community diversity. Correlation networks can provide useful information on the intrinsic characteristics of microbial communities as well as the ecological processes driving community assembly (23). So far, the impacts of ecological processes on microbial interactions are poorly described. A previous study found that microbes have more complicated associations in highly heterogeneous environment (44). Consistent with this, we discovered that associations within the prokaryotic network increased dramatically as heterogeneous selection became a more dominant assembly process in winter, when environmental heterogeneity was the highest (Fig. 6b). Moreover, our findings showed significant seasonal patterns of microbial associations: in cold seasons, prokaryotes became increasingly connected with one another (Fig. 5b, Table S4). The lowest associations in the network were observed in summer when the roles of heterogeneous selection were the least important. Interestingly, prokaryotic β-diversity showed similar trends with the co-occurrence networks (Fig. 6b and c), which were in accordance with previous studies (23, 45). Drift, however, may be linked to fewer network connections as well as a decrease in β-diversity. Thus, we synthesized that temperature can significantly influence environmental heterogeneity, as low temperatures led to high environmental heterogeneity, which then resulted in strong selection processes, especially heterogeneous selection (Fig. 6c). The high ratio of selection processes then made the microbes more interconnected than they were at a low ratio. To the best of our knowledge, this is the first study to establish a clear relationship between microbial seasonal interactions and ecological processes in eutrophic waters. Admittedly, this may be an atypical case in this eutrophic bay, and future studies on different ecosystems will expand this conception.

Temperature clearly had a direct and considerable impact on microbial interactions, as revealed by negative correlations with network topological properties (Fig. 5d), indicating that there were more interconnections at low temperatures in winter. Prokaryotes may be able to exchange metabolites needed for growth more often in cold seasons, which is supported by the largest ratio of positive correlations in winter (Table S4). The predominance of positive correlations indeed suggests the coexistence of prokaryotes with niche and fitness differences in the metacommunity (46), which subsequently leads to the formation of complex interaction networks (47). In the winter, we also found sufficient potential keystone species. Keystone species have been shown to have a considerable influence on other members of the community and to play disproportionately important roles in network structure maintenance (48). Most keystone species in our study were identified as *Alphaproteobacteria* and *Gammaproteobacteria*, and they also dominated the network modules (Fig. 5).

Interactions among microbes can significantly impact community variations, leading to high proportions of unexplained variation that cannot be quantified by VPA (21, 49). *Diatomea* and *Chlorophyta* were the main microeukaryotes affecting variation in the PCCs, especially the *Flavobacteriales* and *Rhodobacterales* community members (Fig. S8). Diatom–bacteria interactions usually comprise higher-level microbial interactions across interdomain networks in marine environment (50). In our study, diatoms were most frequently linked with *Flavobacteriales* and *Rhodobacterales*, which were known to utilize diatom-derived organic matter (2, 25). These heterotrophic bacteria can co-exist with phytoplankton by using dissolved organic matter to maintain their growth, while phytoplankton depend on bacteria-produced nutrients and other substances (e.g., essential minerals, hormones, and vitamins) (25, 51, 52). These mutualistic diatom–bacteria interactions may be enhanced by warm temperatures (53), especially during seasonal diatom blooms (13). *Chlorophyta* were also highly abundant in Xiangshan Bay, and members of this phylum can produce hydrocarbons and exopolysaccharides that provide a nutrient-rich habitat for surrounding prokaryotes (54). Overall, our findings support the idea that PCCs in eutrophic bays are driven in part by the presence of microeukaryotes.

### Conclusions.

Our results clearly demonstrated that assembly of prokaryotes in eutrophic waters were primarily controlled by a balance between deterministic processes like heterogeneous selection, and stochastic processes like drift, with the former being more important during low-temperature seasons and the latter during high-temperature seasons. Furthermore, heterogeneous selection could result in high β-diversity and more tightly connected networks, whereas drift may lead to decline in β-diversity and network associations. Microeukaryotes were important biological factors affecting PCC variations, and the interactions between prokaryotes and microeukaryotes may also influence the assemblies of PCCs. Finally, the conceptual model developed based on the findings of this study linked seasonal microbial interactions to ecological processes, which had not been described. This model sheds fresh light on the seasonal dynamics of prokaryotes in eutrophic waters, paving the way for future research to broaden and test this notion in different ecosystems.

## MATERIALS AND METHODS

### Sample collection.

Surface seawater samples (at a depth of 0.5 m) were taken monthly from January to December (with comparable sampling intervals) in 2018, at 13 sites in Xiangshan Bay, yielding a total of 156 (12 months × 13 sites) samples (Fig. S1). About 5 L seawater was collected in a Niskin bottle in each site. The seasons were defined as follows: spring (all samples from March to May), summer (June–August), autumn (September–November), and winter (December–February). The water samples were firstly filtered via a 200-μm mesh to eliminate large cells and particles. After that, approximately 700 mL of seawater was filtered through membranes with a 0.2-μm pore size (47 mm, Millipore, USA). Finally, DNA was extracted from samples by using a Power Soil DNA extraction kit (Mo Bio, CA, USA) according to the kit’s instruction.

For environmental factors, seawater temperature, salinity, and pH were detected on board. Nitrite, ammonium, nitrate, silicate, phosphate, dissolved oxygen (DO), Chl a, and COD levels were tested using standard methods (55) and as reported by Zhang et al. (19). Using all monitored environmental factors, environmental heterogeneity was calculated according to Huber et al. (23). Specifically, for each season, we computed a Euclidean distance matrix based on all environmental factors. The mean values of the dissimilarity between sites of each computed matrix were then determined and utilized as an index of environmental heterogeneity.

### MiSeq sequencing and data processing.

The V4 hypervariable region sequences of the prokaryotic 16S rRNA and microeukaryotic 18S rRNA gene were amplified using the primer pair 515f/806r (56) and 3NDF/V4_euk_R2R (57), respectively. The PCR cycling consisted of predenaturing at 95°C for 3 min followed by 27 amplification cycles (95°C, 30 s; 55°C, 30 s; 72°C for 45 s), and finally, elongation at 72°C for 10 min. Each sample had triplicates, which were pooled before purification. The pooled amplicons were then gel-purified using a TaKaRa purification kit (TaKaRa Bio, Japan), and all purified products were normalized to equimolar amounts. Finally, the prokaryotic and microeukaryotic libraries were sequenced with a paired-end read run (2 × 300 bp) on a MiSeq platform (Illumina Inc., CA, USA).

Raw sequence data of prokaryotes and microeukaryotes were separately processed using the Quantitative Insights into Microbial Ecology v1.9.1 (58) and USEARCH V6.1 (59) pipelines for quality control and chimera removal. Clean prokaryotic and microeukaryotic sequences were clustered separately into OTUs at a threshold of 97% similarity with UCLUST (60) and annotated using the SILVA128 database (61). Prokaryotic OTUs identified as chloroplast, mitochondrion, or unclassified were removed, as were microeukaryotic OTUs classified as metazoan or unclassified. To minimize PCR and sequencing biases, singletons were discarded. Finally, based on the lowest sequencing depth in a single sample, abundances were rarefied to 25,110 and 13,400 sequences per sample for prokaryotes and microeukaryotes, respectively. The final OTU table for prokaryotes contained 31,038 OTUs. Because microeukaryotic data were largely used for correlation analysis, they are not discussed in depth here.

### Statistical analyses.

The correlations among the top 10 abundant phyla and environmental factors were determined by Spearman’s rank correlation and visualized using the pheatmap R (version 3.3.3) package (62). The top 50 most abundant OTUs were selected to clarify the seasonality of PCCs, which was then shown by using the pheatmap R package (62). The Spearman’s rank correlations were performed to evaluate the relationships between the 50 most abundant OTUs and environmental factors, and then the significant (FDR-adjusted *P* value < 0.01) and robust (Spearman’s |ρ| > 0.6) correlations were retained and visualized by Gephi network (63).

The taxonomic β-diversity (Bray-Curtis distance) was illustrated by nonmetric multidimensional scaling (NMDS) plots. Differences in PCCs across the four seasons were computed using permutational multivariate analysis of variance (PERMANOVA) and multiple-response permutation procedure (MRPP) based on the Bray-Curtis distance using the vegan R package (64). The correlations between community variation (Bray-Curtis distance) and environmental factors (Euclidean distance) were also assessed using Mantel tests (65). Environmental factors that have a substantial influence on prokaryotic β-diversity were fitted to the NMDS plot using the vegan package’s “envfit” function (64). Fits for temperature (Euclidean distance) and microeukaryotic communities (MECs, Bray-Curtis distance) with PCCs (Bray-Curtis distance) were carried out with the “lm” function in the ggplot2 R package (66). The β-diversity (Bray-Curtis distance) of the major prokaryotes at the phylum/class level was also fitted with the MECs (Bray-Curtis distance) using the “lm” function in the ggplot2 R package (66).

### Partitioning the environment and geographic distance effects.

A redundancy analysis-based VPA was utilized to test the proportional contributions of environmental factors and geographic distance to PCC variations (67). Environmental factors and geographic distance were forward-selected prior to the VAP analysis using redundancy analysis and principal coordinates of neighbor matrices (PCNM) (68). The PCNM variables were calculated using a principal coordinate analysis (PCoA) on the shortened distance matrix connecting all sites. Then, the PCNM variables were selected in a forward selection using the reported method (20, 69). The selected environmental factors, linear trend factors, and PCNM variables were then produced, and all nonsignificant variables were removed from the subsequent analyses. Finally, variation partitioning was calculated for the PCCs between the selected environmental factors, linear trend factors, and PCNM variables using the vegan’s “varpart” function (64).

A partial least-squares path model (PLS-PM) was utilized to investigate the direct and indirect connections among the environmental factors, PCCs, and MECs using the plspm R package (70). The physicochemical factors included pH, salinity, COD, and Chl a, and the nutrients included phosphate, silicate, ammonium, nitrite, and nitrate. Longitude and latitude were selected as geographic factors. Richness, evenness, phylogenetic diversity, and Shannon index were utilized to define the microeukaryotic α-diversity. The microeukaryotic β-diversity was represented by the NMDS axis2 (NMDS2) based on the Bray-Curtis distance, while prokaryotic β-diversity was represented by the NMDS axis1 (NMDS1).

### Quantification of ecological processes structuring the PCCs.

Ecological processes were classified and quantified as reported by Stegen et al. (43). Briefly, the weighted β-nearest taxon index (βNTI) (42, 71) was computed and used to divide the OTUs into two pairwise communities based on the standard deviation of the phylogenetic distances from the null model. Then, the ecological processes driving the PCCs was further identified by βNTI together with the Raup-Crick (RC_bray_) metric using Bray–Curtis distance (43). After that, we defined heterogeneous selection as the fraction with a βNTI value of > +2 and homogeneous selection as a βNTI value of < −2 (43). In addition, |βNTI| < 2 and |RC_bray_| < 0.95 suggest that drift (undominated processes) acts alone in shaping community assembly (43). However, |βNTI| < 2, but RC_bray_ < −0.95 (RC_bray_ > +0.95), suggests that community assembly was driven by homogenizing dispersal (dispersal limitation) (43).

### Co-occurrence patterns of prokaryotic communities.

To elucidate the prokaryotic intradomain co-occurrence patterns, OTUs with more than 10 sequences in all samples were kept for further analyses. The correlations among the prokaryotic OTUs in each season were calculated using the SparCC algorithm implemented in the SparCC Python module with default settings (72). Only significant (*P* value < 0.05) and robust (SparCC |r| > 0.5) correlations were retained for further co-occurrence network analyses, and a GML file for each network was created using the igraph R package (73). Meanwhile, network-level topological features (i.e., average path length, average clustering coefficient, and diameter) were computed. Visualization of networks was then implemented in Gephi (63), and modular analysis was further carried out based on Louvain algorithm (74). The real network for each season was compared with its relative 1000 Erdös-Réyni random network (75), which had the equal number of nodes and edges as the real networks. Keystone species were chosen as the nodes with low betweenness centrality values (< 5,000) and high degree (> 100) in four co-occurrence networks. The subnetworks for each sample of the four seasons were created from meta-community networks by retaining the OTUs occurred in each sample using the igraph R package (73). Meanwhile, network degree, betweenness centrality, and transitivity were computed for each subnetwork, and the relationships between temperature and them were fitted by “lm” function in the ggplot2 R package (66).

To estimate the influence of microeukaryotes on PCCs, Spearman’s rank correlation was utilized to compute correlations between the top 50 most abundant microeukaryotic and prokaryotic OTUs, and only significant correlations (FDR-adjusted *P* value < 0.05) were kept for further analysis. Using the bipartite R package, the obtained correlation network was converted to the matrices 1 or 0 to show the presence or absence, respectively, of the corresponding prokaryote–microeukaryote associations and visualized with a bipartite graph (76).

### Data availability.

All sequence data were deposited in the NCBI Sequence Read Archive with the BioProject ID PRJNA756123 for prokaryotes.
