# Supplementary material for: Disentangling the Mechanisms Shaping the Prokaryotic Communities in a Eutrophic Bay
Source: Microbiol Spectr. 2022 May 31;10(3):e01481-22. doi: 10.1128/spectrum.01481-22 (PMC9241920; doi:10.1128/spectrum.01481-22)
Supplement: SUPPLEMENTAL FILE 1 — Supplemental material. Download spectrum.01481-22-s0001.pdf, PDF file, 6.5 MB [file spectrum.01481-22-s0001.pdf]

Disentangling the mechanisms shaping the prokaryotic communities in a eutrophic bay

Huajun Zhang [a,b]*, Yi Yan [a,b], Tenghui Lin [a,b], Weijuan Xie [b], Jian Hu [a,b], Fanrong Hou [b], Qingxi Han [a,b], Xiangyu Zhu [c], Demin Zhang [a,b]*

[a] State Key Laboratory for Managing Biotic and Chemical Threats to the Quality and Safety of Agro-products, Ningbo University, Ningbo, 315211, China

[b] Key Laboratory of Applied Marine Biotechnology of Department of Education, Ningbo University, Ningbo, 315211, China

[c] Environmental Monitoring Center of Ningbo, Ningbo, 315010, China

* For correspondence, E-mail: zhanghuajun@nbu.edu.cn (Huajun Zhang) or zhangdemin@nbu.edu.cn (Demin Zhang).

Table S1 Average relative abundance of prokaryotes in the four seasons

	Winter	Spring	Summer	Autumn
Alphaproteobacteria	0.309^{a}	0.405^{b}	0.371^{bc}	0.320^{ac}
Gammaproteobacteria	0.170^{a}	0.161^{ab}	0.133^{b}	0.172^{a}
Marine Group I	0.103^{a}	0.060^{b}	0.087^{ab}	0.143^{c}
Flavobacteriia	0.068^{a}	0.138^{b}	0.084^{a}	0.064^{a}
Betaproteobacteria	0.117^{a}	0.093^{b}	0.038^{c}	0.055^{c}
Acidimicrobiia	0.046^{a}	0.048^{a}	0.090^{b}	0.065^{a}
Cyanobacteria	0.001^{a}	0.001^{a}	0.096^{b}	0.046^{c}
Thermoplasmata	0.018^{a}	0.022^{ab}	0.035^{b}	0.032^{ab}
Actinobacteria	0.022^{a}	0.030^{b}	0.013^{c}	0.008^{c}
Deltaproteobacteria	0.021^{a}	0.002^{b}	0.009^{bc}	0.016^{ac}
Sphingobacteriia	0.004^{a}	0.005^{a}	0.006^{a}	0.017^{b}
Planctomycetacia	0.009^{a}	0.004^{b}	0.005^{ab}	0.008^{a}
Bacilli	0.010^{a}	0.007^{a}	0.003^{a}	0.004^{a}

Note: Abundance with totally different superscript letters indicate significant difference ($P < 0.05$).

Table S2 Dissimilarity test showing the difference of prokaryotic composition between each of two compared seasons

	MRPP		PERMANOVA	
	Delta	*P*	F	*P*
Spring vs Summer	0.420	0.001	36.5	0.001
Spring vs Autumn	0.436	0.001	40.8	0.001
Spring vs Winter	0.425	0.001	14.6	0.001
Summer vs Autumn	0.432	0.001	10.3	0.001
Summer vs Winter	0.479	0.001	20.2	0.001
Autumn vs Winter	0.495	0.001	14.2	0.001

MRPP: multiple-response permutation procedure; PERMANOVA: permutational multivariate analysis of variance.

Table S3 Mantel tests showing the correlations between environmental factors (Euclidean distance) and prokaryotic β-diversity (Bray–Curtis distance) with 999 permutations

	**Spring**		**Summer**		**Autumn**		**Winter**	
	ρ	*P*	*ρ*	*P*	*ρ*	*P*	*ρ*	*P*
Temperature	0.592	0.001	0.597	0.001	0.326	0.001	0.251	0.001
Microeukaryote	0.349	0.001	0.571	0.001	0.405	0.001	0.349	0.001
Salinity	0.366	0.001	0.388	0.001	0.313	0.001	0.278	0.001
pH	0.306	0.001	0.214	0.002	0.309	0.001	0.221	0.017
COD	0.263	0.002	0.204	0.069	0.279	0.002	0.120	0.071
Chl a	-0.058	0.807	0.285	0.001	0.141	0.069	0.034	0.330
Ammonium	0.150	0.048	0.251	0.006	0.181	0.058	0.178	0.026
Nitrite	0.153	0.010	0.271	0.001	0.008	0.192	0.227	0.003
Nitrate	0.171	0.014	0.292	0.001	0.272	0.001	0.114	0.064
Phosphate	0.105	0.071	0.217	0.003	0.301	0.001	0.039	0.236
Silicate	0.452	0.001	0.201	0.001	0.204	0.006	0.052	0.231

Note: COD, chemical oxygen demand; DO, dissolved oxygen; Chl a, chlorophyll a.

Table S4 Topological features of the real co-occurrence networks in each season and their relevant random networks

	Real networks							Random networks		
	Nodes	Edges	Modularity	Average clustering coefficient	Network diameter	Average path length	Average degree	Modularity (SD)	Network diameter	Average path length (SD)
Winter	855	17790 <14709>	0.409	0.656	8	2.87	41.6	0.093 (±0.002)	3	2.08 (± 0.0006)
Spring	273	3297 <1920>	0.316	0.581	7	2.70	24.2	0.141 (±0.006)	3	2.018 (± 0.002)
Summer	299	3240 <2014>	0.324	0.605	6	2.59	21.7	0.152 (±0.006)	3	2.12 (± 0.002)
Autumn	470	5498 <3598>	0.320	0.643	7	2.74	23.4	0.144 (±0.004)	3.05	2.25 (± 0.001)

Note: Numbers in angle brackets indicate the number of positive edges.

Table S5 Number of connections between prokaryotes and micreukaryotes in the bipartite network.

	Chlorophyta	Cryptomonadales	Diatomea	Syndiniales	Dinoflagellata	Alveolata_others	Cercozoa	Ciliophora	Opisthokonta	Picozoa	Prymnesiales	Rhizaria
Acidimicrobiales	31	10	27	15	9	8	4	7	2	3	4	3
Flavobacteriales	69	24	66	33	21	16	9	10	7	6	7	5
Oceanospirillales	28	17	29	13	8	12	4	3	4	2	3	1
Rhodobacterales	66	28	64	27	14	16	11	7	8	5	5	5
Rhodospirillales	11	2	10	5	2	3	1	0	1	1	1	1
SAR11	30	7	31	13	5	5	3	3	3	3	3	3
SAR116	19	10	17	5	2	4	2	2	2	1	2	2
Lactobacillales	3	0	8	2	3	1	2	1	1	1	0	0
Micrococcales	12	10	18	9	6	6	2	2	2	0	1	0
Burkholderiales	10	4	9	4	1	3	1	1	1	1	1	1
Hydrogenophilales	6	5	9	3	2	1	1	1	1	0	1	1
Methylophilales	8	6	9	6	3	3	1	2	1	1	1	1
Prochlorococcus	8	5	9	6	3	3	1	1	1	1	1	1
Synechococcus	8	6	9	6	2	3	1	2	1	1	1	1
Cyanobacteria_others	8	5	9	6	3	3	1	1	1	1	1	1
Cellvibrionales	8	2	10	6	3	4	2	1	1	1	1	1
Pseudomonadales	6	1	2	2	4	3	1	0	1	1	0	1
Xanthomonadales	8	4	2	3	3	3	1	2	0	1	0	0
γ-proteobacteria_others	9	4	9	3	1	1	1	1	1	1	1	1
Sphingobacteriales	10	5	9	5	3	3	2	1	1	1	1	1
Marine Group I	12	6	12	7	6	6	2	3	2	0	1	1
Marine Group II	14	7	14	4	2	3	2	2	2	2	1	0

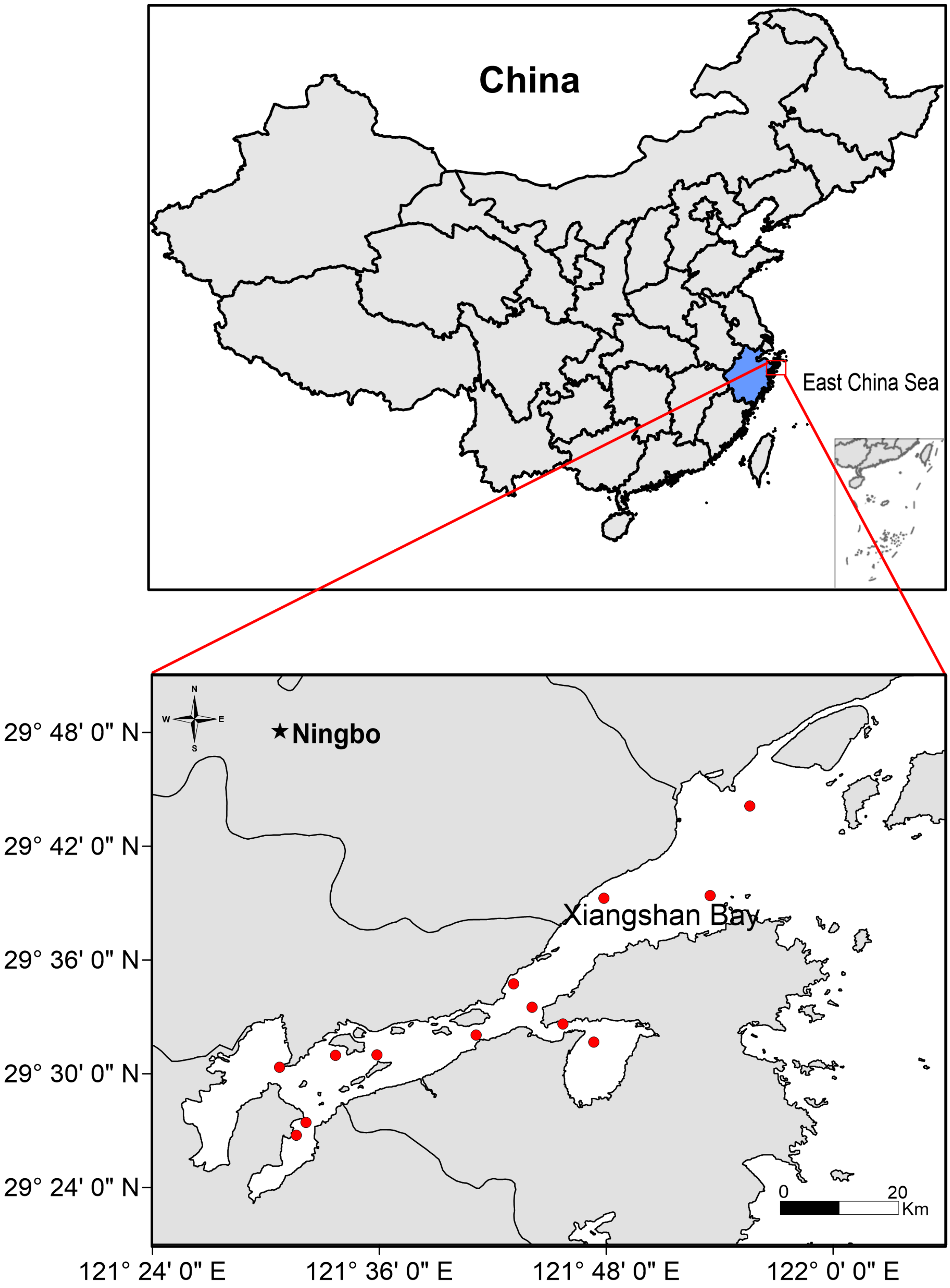

Figure S1. Locations of the sampling sites. Xiangshan bay is in the Zhejiang province (Blue area). A total of 156 surface seawater samples were collected monthly from January to December 2018 at 13 sites (Red dots).

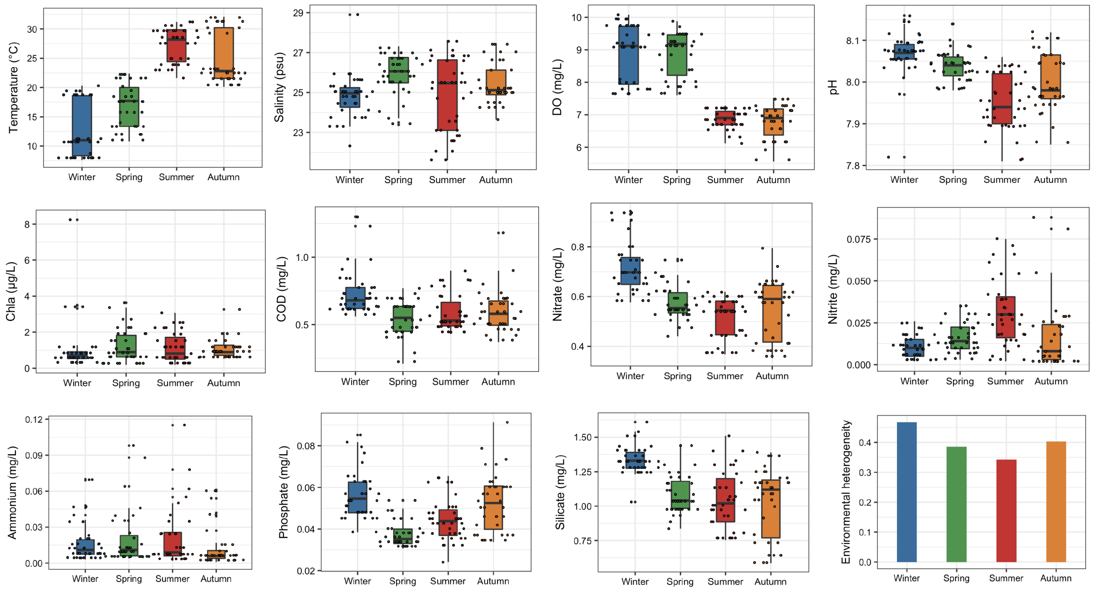

Figure S2. Changes of environmental factors in the four seasons. The last panel is environmental heterogeneity. COD: chemical oxygen demand; DO: dissolved oxygen; Chl a: chlorophyll a.

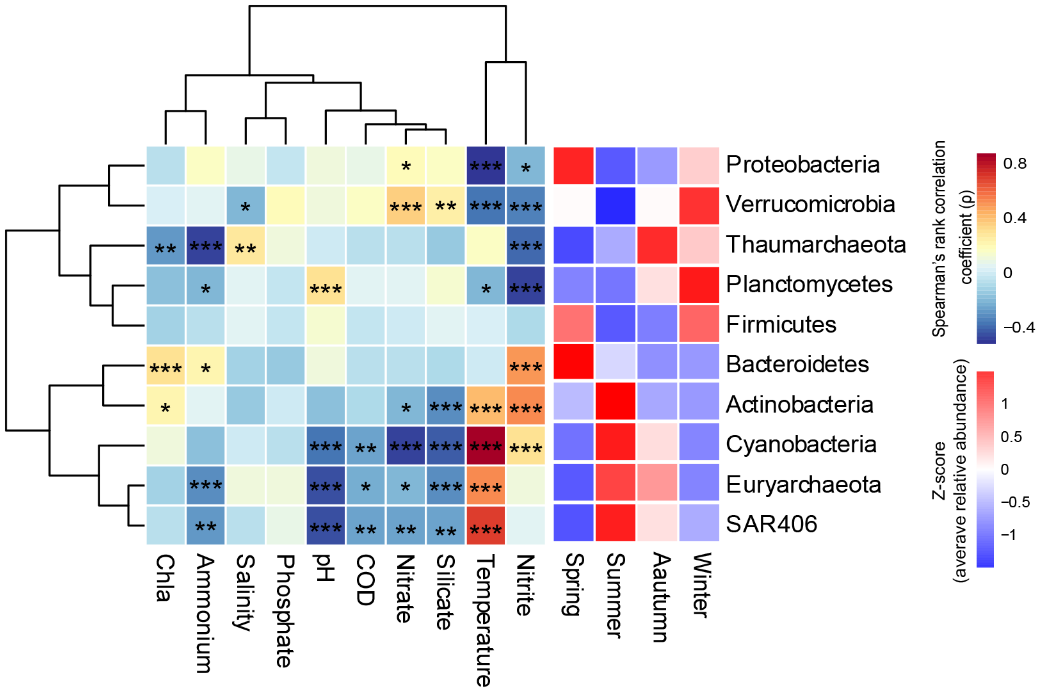

Figure S3. Relative abundance of 10 dominant phyla (right panel) and their correlations with environmental factors (left panel).

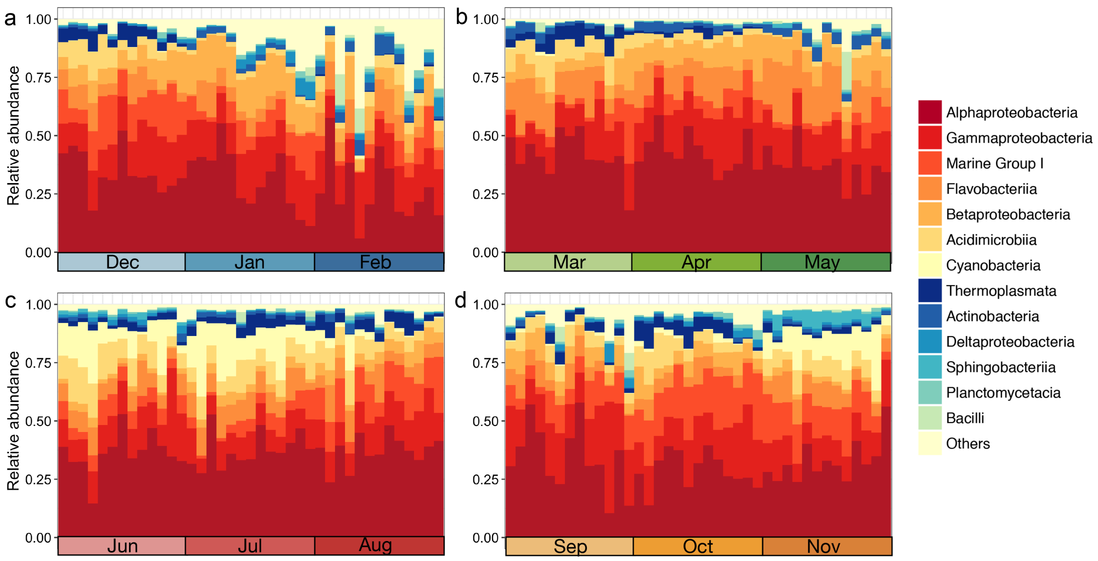

Figure S4. Relative abundance of prokaryotes at the class level in the four seasons. a-d: Winter, Spring, Summer and Autumn, respectively.

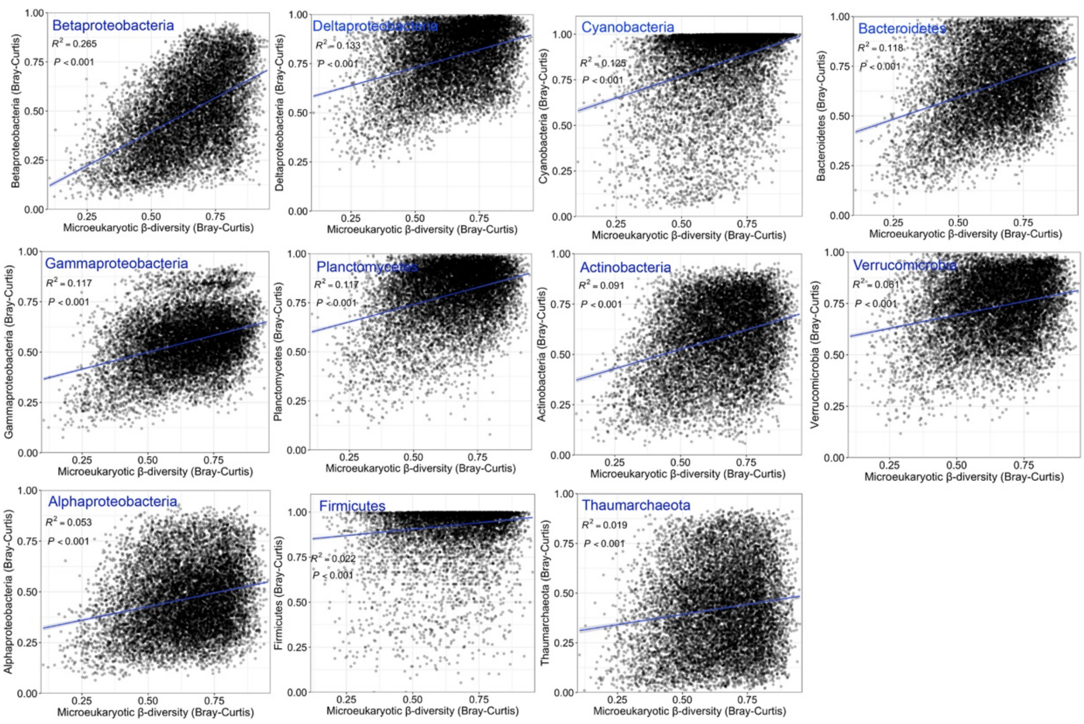

Figure S5. Relationship between prokaryotes and microeukaryotes.

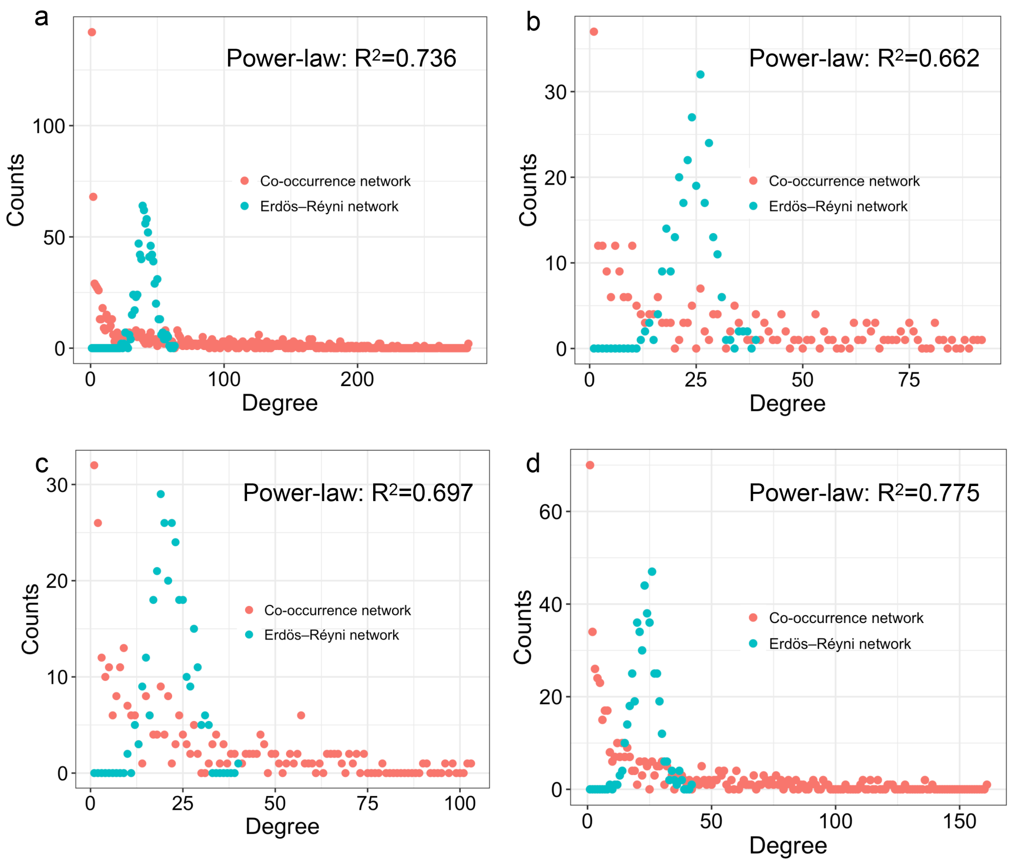

Figure S6. The distribution of degree for real co-occurrence network (Red) and Erdös-Réyni random network (Blue) in the four seasons. a-d: Winter, Spring, Summer and Autumn, respectively.

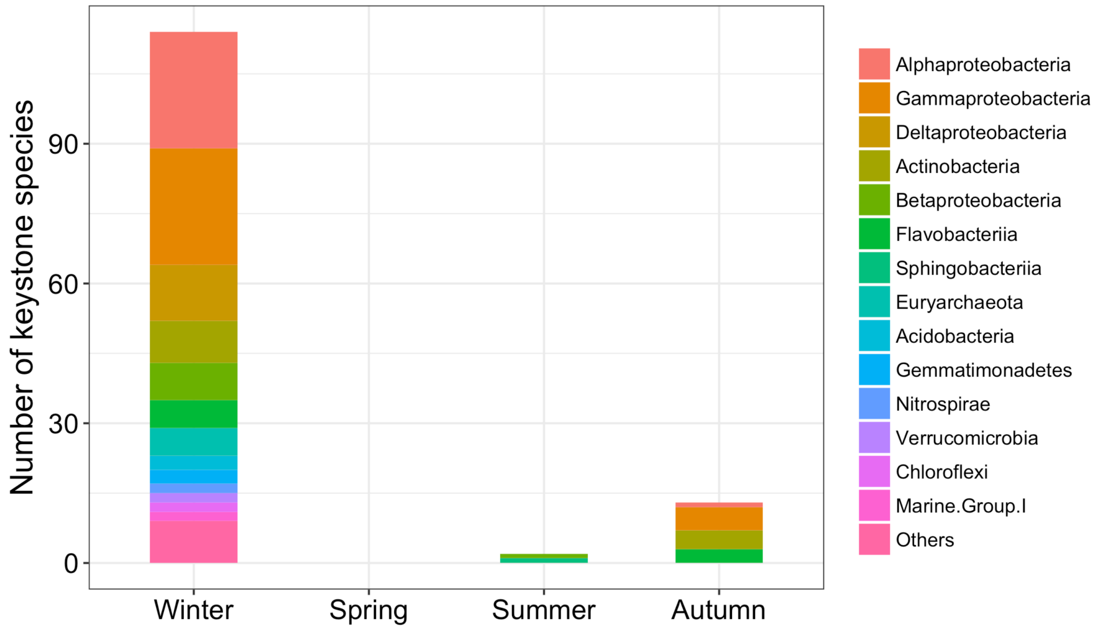

Figure S7. Number of keystone species in the four seasons.

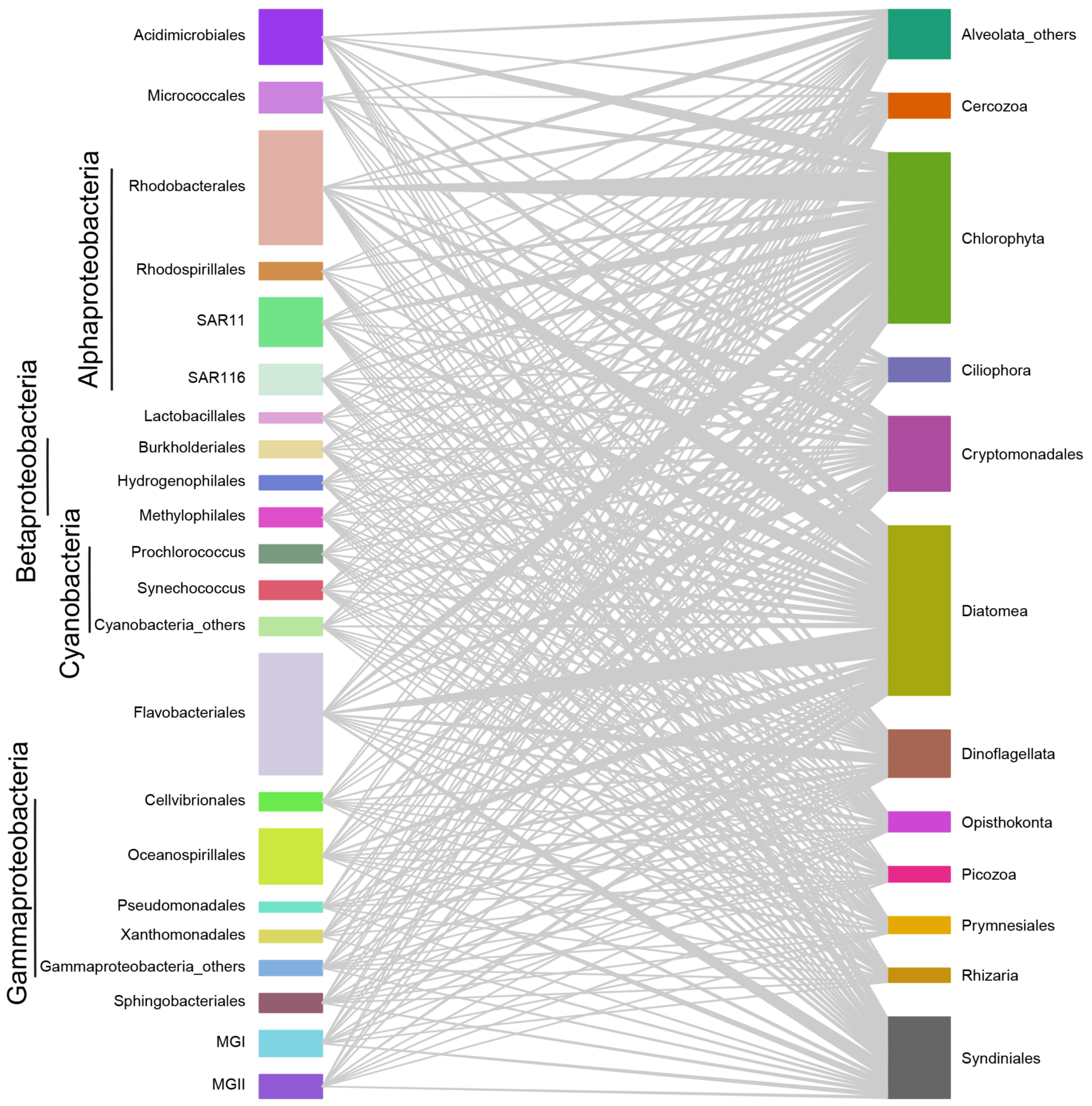

Figure S8. Bipartite network analysis prokaryotic-microeuakryotic interactions.
